# Change in diffusion weighted imaging after induction chemotherapy outperforms RECIST guideline for long-term outcome prediction in advanced nasopharyngeal carcinoma

**DOI:** 10.1186/s40644-025-00854-4

**Published:** 2025-03-12

**Authors:** Qi Yong H. Ai, Ho Sang Leung, Frankie K.F. Mo, Kaijing Mao, Lun M. Wong, Yannis Yan Liang, Edwin P. Hui, Brigette B.Y. Ma, Ann D. King

**Affiliations:** 1 Department of Diagnostic Radiology, The University of Hong Kong, Pokfulam, Hong Kong S.A.R. P.R. China; 2 Department of Health Technology and Informatics, The Hong Kong Polytechnic University, Kowloon, Hong Kong S.A.R P.R. China; 3 Department of Imaging and Interventional Radiology, The Chinese University of Hong Kong, Prince of Wales Hospital, Shatin, New Territories, Hong Kong S.A.R P.R. China; 4 Department of Clinical Oncology, State Key Laboratory of Translational Oncology, Sir Y.K. Pao Centre for Cancer, The Chinese University of Hong Kong, Prince of Wales Hospital, Shatin, Hong Kong S.A.R P.R. China; 5 Restorative Dental Sciences, Faculty of Dentistry, The University of Hong Kong, Pokfulam, Hong Kong SAR China

**Keywords:** Head and neck cancer, Diffusion weighted imaging, Outcome prediction, Nasopharyngeal carcinoma, RECIST guideline

## Abstract

**Purpose:**

To investigate change in diffusion weighted imaging (DWI) between pre-treatment (pre-) and after induction chemotherapy (post-IC) for long-term outcome prediction in advanced nasopharyngeal carcinoma (adNPC).

**Materials and methods:**

Mean apparent diffusion coefficients (ADCs) of two DWIs (ADC_pre_ and ADC_post−IC_) and changes in ADC between two scans (ΔADC%) were calculated from 64 eligible patients with adNPC and correlated with disease free survival (DFS), locoregional recurrence free survival (LRRFS), distant metastases free survival (DMFS), and overall survival (OS) using Cox regression analysis. C-indexes of the independent parameters for outcome were compared with that of RECIST response groups. Survival rates between two patient groups were evaluated and compared.

**Results:**

Univariable analysis showed that high ΔADC% predicted good DFS, LRRFS, and DMFS *p* < 0.05), but did not predict OS (*p* = 0.40). Neither ADC_pre_ nor ADC_post−IC_ (*p* = 0.07 to 0.97) predicted outcome. Multivariate analysis showed that ΔADC% independently predicted DFS, LRRFS, and DMFS (*p* < 0.01 to 0.03). Compared with the RECIST groups, the ΔADC% groups (threshold of 34.2%) showed a higher c-index for 3-year (0.47 vs. 0.71, *p* < 0.01) and 5-year DFS (0.51 vs. 0.72, *p* < 0.01). Compared with patients with ΔADC%<34.2%, patients with ΔADC%≥34.2% had higher 3-year DFS, LRRFS and DMFS of 100%, 100% and 100%, respectively (*p* < 0.05).

**Conclusion:**

Results suggest that ΔADC% was an independent predictor for long-term outcome and was superior to RECIST guideline for outcome prediction in adNPC. A ΔADC% threshold of ≥ 34.2% may be valuable for selecting patients who respond to treatment for de-escalation of treatment or post-treatment surveillance.

**Supplementary Information:**

The online version contains supplementary material available at 10.1186/s40644-025-00854-4.

## Introduction

Nasopharyngeal carcinoma (NPC) is sensitive to radiotherapy (RT) and can be cured by only RT when it is diagnosed at early stage with a favorable 5-year disease free survival (DFS) of over 90% [1, 2]. However, early-stage NPCs are usually overlooked as they rarely cause typical symptoms and so over 70% of patients have advanced NPC (adNPC) at diagnosis [1]. Patients with adNPC have disease with a greater likelihood of resistance to treatment and so the treatment of choice is now induction chemotherapy (IC) before the course of concurrent chemoradiotherapy (CCRT) [3]. However, there still have about 30% of these patients eventually develop disease recurrence after treatment [4]. The additional IC and full course of the CCRT increase risks of treatment-related toxicity, which is now one of the major causes of the decreased quality of life and even death after treatment. Therefore, early prediction of risk of recurrences in patients with adNPC would be beneficial because additional immunotherapy or targeted therapy and close post-treatment surveillance can be timely intervened for patients at high risk of disease recurrences while de-escalation of chemotherapy may be applied to patients at low risk of disease recurrence.

Pre-treatment MRI is the long-established imaging modality of choice for staging disease in the head and neck and the addition of diffusion weighted imaging (DWI), which is a short sequence, is easily accommodated in the MRI protocol [5, 6]. DWI can measure the random Brownian motion of water molecules within tissues. In previous head and neck squamous cell carcinoma (HNSCC) studies, DWI has shown the potential for the prediction of long-term post-treatment outcome [7–21]. Of many DWI parameters, change in mean values of apparent diffusion coefficient (ΔADC%) between pre- and intra-treatment scans is the most promising one for clinical practice because ΔADC% is less influenced by variability among scanners, techniques and scanning protocols. In NPC, the recent move to add upfront chemotherapy before CCRT requires a second MRI scan after tumor shrinkage to plan the radiotherapy field. This provides the opportunity to perform a second DWI examination to monitor treatment change in the tumor microenvironment. Although many studies have showed ΔADC% is valuable for the prediction of short-term outcome [22–24], there is little literature reported whether ΔADC% can also predict long-term outcome in NPC [25].

Therefore, in this study, we aimed to investigate the predictive value of DWI on the pre-treatment and post-IC scans and change in DWI after IC for long-term outcome in adNPC by correlating the mean values of ADC on the pre- and post-IC scans (ADC_pre_ and ADC_post−IC_) and ΔADC% with long-term outcome. Furthermore, we compared the predictive values of the DWIs with that of treatment response to IC detected by anatomical change in tumour size using the internationally widely used RECIST guideline [26].

## Materials and methods

### Patients

This retrospective study was approved by the local institutional ethics review board, and the requirement for written consent was waived for this retrospective study. All study procedures complied with the tenets of the Declaration of Helsinki. Patients who fulfilled the following inclusion criteria were included in the study: (1) ethnic Chinese adult patients with new biopsy-proven NPC; (2) patients who had pre-treatment staging head and neck MRI from 2014 to 2022 showing stage III or stage IVa NPC in our institution; (3) patients treated with 2–3 cycles of IC followed by CCRT; and (4) patients who had a post-IC head and neck MRI (post-IC MRI) including DWI of the primary tumour using the same DWI protocol at both time points (pre- and post-IC MRIs). Patients were excluded from the analysis if: (1) patient was lost to follow-up; (2) MR images were severely degraded by motion artifacts or other artifacts; or (3) patient who had a history of another head and neck cancer or secondary primary tumours treated with different regimens. Some of the patients in this study was previously reported by Kwong et al. [27].

### Image acquisition

MRI was performed on a Philips Achieva TX 3 T scanner (Philips Healthcare, Best, The Netherlands) or a GE 3 T scanner (GE HealthCare, Chicago, United States) with body coils for radiofrequency transmission and a 16-channel Philips neurovascular phased-array coil for reception. Patients underwent a pre-treatment MRI and post-IC MRI with DWI and anatomical imaging.

DWI was acquired using a fat-suppressed, single-shot spin-echo echo-planar imaging sequence. The imaging parameters were: repetition time/echo time, 2000/50 mesc; field of view, 230 mm × 230 mm; resolution, 1.7 mm × 2.1 mm; slice thickness, 4 mm; number of slices: 9; echo train length, 55; sensitivity encoding factor, 2; number of signals acquired, 4 and at least 2 b-values (0 and 1000 s/mm^2^).

Anatomical MRI sequences included at minimum of (1) axial non-contrast-enhanced fat-suppressed T2-weighted images (non-CE FS-T2WI), (2) axial non-CE T1-weighted images (non-CE T1WI), and (3) axial contrast-enhanced with or without FS-T1WI.

### Imaging analysis

#### DWI analysis

Olea Sphere (version 3.0; Olea Medical SA) was used for the diffusion post-processing steps by implementing a Bayesian probability-based algorithm using two b-values (0 and 1000 s/mm^2^) to fit a mono-exponential diffusion model to calculate the conventional apparent diffusion coefficient (ADC).

The primary tumour on the pre- and post-IC ADC maps was contoured manually excluding any necrotic or cystic areas with reference to the corresponding anatomical images by a researcher with 10 years’ experience in MRI of NPC. The mean values of pre- and post-IC ADC (ADC_pre_ and ADC_post−IC_), and change in ADC between pre- and post-IC scans, which was defined as ΔADC% = [(ADC_post−IC_ - ADC_pre_)/ ADC_pre_ *100%] was calculated for further analysis. Intra- and inter-observer analyses for DWI were not analysed as previous NPC studies have shown the ICCs for ADC of > 0.90 [28–31].

#### Evaluation of treatment response to IC based on RECIST 1.1 guideline [26]

Treatment response to IC was evaluated according to the RECIST 1.1 criteria, using change in the maximum diameters of the primary NPC. Treatment response to IC was categorized into (1) complete response (CR), defined as disappearance of primary tumour, (2) partial response (PR), defined as at least 30% reduction in the maximum diameters of primary NPC, (3) progressive disease (PD), defined as at least 20% increase in the maximum diameters of primary NPC, or (4) stable disease (SD), defined as insufficient increase or reduction in the maximum diameters of primary NPC. Patients were then divided into a response group (CR or PR) and a non-response group (SD or PR) for the analysis.

### Treatment

All patients were treated with IC followed by CCRT. The IC was administered intravenously once every 3 weeks for 2 or 3 cycles, with one of the IC regimens: (1) 1000mg/m^2^ of body surface area (BSA) of gemcitabine on day 1 and 8 along with 80 mg/m^2^ of body surface area of cisplatin on day 1, (2) with 175 mg/m^2^ of body surface area of paclitaxel and 75mg/m^2^ of body surface area of cisplatin on day 1; (3) with 75mg/m^2^ of body surface area of docetaxel and 75mg/m^2^ of body surface area of cisplatin on day 1, or (4) with 70 mg/m^2^ of body surface area of paclitaxel on day 1, 8, and 15 and carboplatin area under the concentration-time curve (AUC) 5–6 mg/ml.min on day 1. The CCRT was given by administering 40 mg/m^2^ of body surface area of cisplatin weekly or carboplatin target AUC of 2 mg/ml.min weekly for up to 7 cycles intravenously, concurrently with 66–74 Gy of radiation to the primary tumour and enlarged lymph nodes, and 50–60 Gy of radiation to regions at risk of microscopic spread given in 33–35 fractions.

### Patient follow-up and endpoints

All patients underwent regular follow-up after treatment once every 3 months for the first 12 months, every 6 months for the next 24 months, and once yearly afterwards until diagnosis of recurrence or death. The disease-free survival (DFS), locoregional recurrence-free survival (LRRFS), distant metastasis-free survival (DMFS), and overall survival (OS) were calculated from the date of the start of treatment to the date of any disease recurrence, locoregional recurrence, distant metastasis, and last date of follow-up or date of death, respectively.

### Statistical analysis

Difference in ADC values between pre- and post-IC scans was evaluated using the paired student t-test. The diffusion weighted parameters (ADC_pre_, ADC_post-IC_, and ΔADC%) were correlated with DFS, LRRFS, DMFS and OS using univariable Cox regression. Significant parameters, together with age, sex, and T-category, N-category, overall stage, cycles of IC and cycles of concurrent chemotherapy were then added into multivariable Cox regression to identify the independent parameters for the prediction of outcome. Receiver-operating characteristic curve analysis and area under the curve (AUC) calculations of significant variables were used to identify the optimal thresholds by maximising the sensitivity plus specificity for the prediction of disease recurrence. The predictive performances of the independent parameters using the optimal thresholds for 3-year and 5-year DFS, LRRFS, DMFS and OS were compared with that of RECIST response groups using concordance statistics by the methods of both Harrel et al. and Uno et al. [32, 33]. The method of Harrel et al. provides an overall measure of differences, and the method of Uno et al. estimates differences from baseline to a specific time point [34], with 1000 bootstrapping to provide the biased-corrected c-index and corresponding 95% confidence intervals (CIs) [32, 35]. The survival rates between two groups of patients were evaluated using the Kaplan-Meier analysis, and differences in survival rates between two groups of patients were compared by log-rank test. A two-sided p-value of < 0.05 indicated statistical significance. All analyses were performed using the SPSS (24.0 version, IBM, NY, USA) statistical analysis software, SAS (9.4 version, SAS Institute Inc., Cary, NC).

### Sample size calculation

According to our previous study [27], we assumed a 3-year DFS of at least 90% for the low risk group and a 3-DFS of lower than 65% for the high risk group, and the median DFS for low risk group was around 48 months in this study. In order to detect the 25% different of 3-year DFS with one-sided alpha level of 0.05 and power of 80%, at least 64 patients were required.

## Results

### Patients

There were 64 patients eligible for the analysis. The patient demographics, T- and N-categories, diffusion weighted parameters (ADC_pre_, ADC_post−IC_, and ΔADC%), RECIST response groups, treatment details, outcome, and length of follow-up time are shown in Table 1.

**Table 1:** Patient demographics, cancer staging, measurements, RECIST groups, and outcome

Characteristic	Numbers of patients (%)
Age	
Median age (range) (years)	54 (25 to 74)
**Sex**	
Male	51 (79.7%)
Female	13 (20.3%)
**T-category**	
T1	9 (14.1%)
T2	3 (4.7%)
T3	28 (43.7%)
T4	24 (37.5%)
**N-category**	
N0	3 (4.7%)
N1	11 (17.2%)
N2	24 (37.5%)
N3	26 (40.6%)
**Overall stage**	
Stage III	22 (34.4%)
Stage IVa	42 (65.6%)
**DWI parameters**	
ADC_pre_ (×10^− 3^ mm^2^/s)	0.82 (0.58 to 1.04)^#^
ADC_post-IC_ (×10^− 3^ mm^2^/s)	1.05 (0.59 to 2.38) ^#^
ΔADC%	28.6% (-8.2%–174.4%) ^#@^
**RECIST response**	
Complete response	4 (6.3%)
Partial response	34 (53.1%)
Stable disease	26 (40.6%)
Progressed disease	0 (0%)
**IC treatment**	
Gemcitabin + cisplatin/ others	60 (93.8%)/ 4 (6.2%)
2 cycles/ 3 cylces	16 (25.0%)/ 48 (75.0%)
**Concurrent chemotherapy**	
Cisplatin/ others	57 (89.1%)/ 7 (10.9%)
Median cycles (range)	6 (2–7)
**Outcomes**	
Disease recurrence	16 (25.0%)
Locoregional recurrence	7 (10.9%)
Distant metastases	11 (17.2%)
Death	11 (17.2%)
**Follow-up**	
Median time (range)(months)	44.7 (16.5 to 111.0)

# indicates data shown as median values (range); @ value calculated by using (pre - post-IC)/pre × 100% and positive value indicates decrease in size after IC

### DWI for the prediction of outcome in patients with adNPC

The ADC_post−IC_ increased in 60/64 (93.8%) (from 0.4 to 174.4%) patients and decreased in 4/64 (6.2%) (from − 8.2% to -0.6%) patients. The difference in ADC values between pre- and post-IC scans was statistically significant (0.81 ± 0.11 vs. 1.10 ± 0.29 × 10^− 3^ mm^2^/s, *p* < 0.01).

There were 16/64 (25.0%) patients with disease recurrence and 48/64 (75.0%) patients without disease recurrence at the end of the follow-up period. The 3-year DFS, LRRFS, DMFS, and OS rates were 78.5%, 89.8%, 83.0%, and 94.6% respectively. The univariable analysis showed that high ΔADC% predicted good DFS (HR = 0.959, 95CI% = 0.932–0.987, *p* < 0.01), LRRFS (HR = 0.932, 95CI% = 0.882–0.986, *p* = 0.01), and DMFS (HR = 0.967, 95CI% = 0.936–0.999, *p* = 0.04), but did not predict OS (*p* = 0.40). Neither ADC_pre_ (*p* = 0.07–0.31) nor ADC_post−IC_ (*p* = 0.10–0.97) predicted survival outcome.

**Fig. 1:**
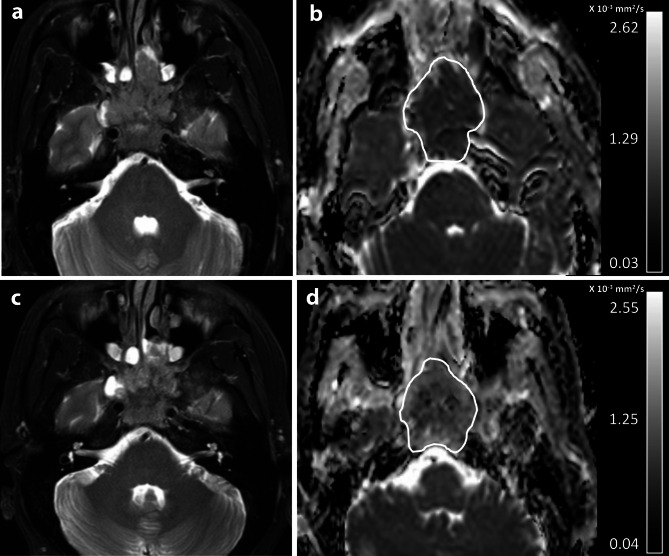
Pre-treatment (**a**-**b**) and post-IC (**c**-**d**) MRIs of a patient with stage T4 NPC that had no recurrence 36 months after treatment. The axial images comprise T2-weighted fat-suppressed images (**a** and **c**) and ADC maps (**b** and **d**). The mean ADC_pre_ and ADC_post−IC_ extracted from the primary NPC (white contours) on the ADC maps were 0.81 × 10^− 3^ mm^2^/s, and 1.14 × 10^− 3^ mm^2^/s, respectively and the ΔADC% was 40.7%. The good long-term outcome was predicted by a high percentage increase in mean ADC value (ΔADC% of ≥ 34.2%) on the post-IC DWI compared with that on the pre-treatment DWI.

**Fig. 2:**
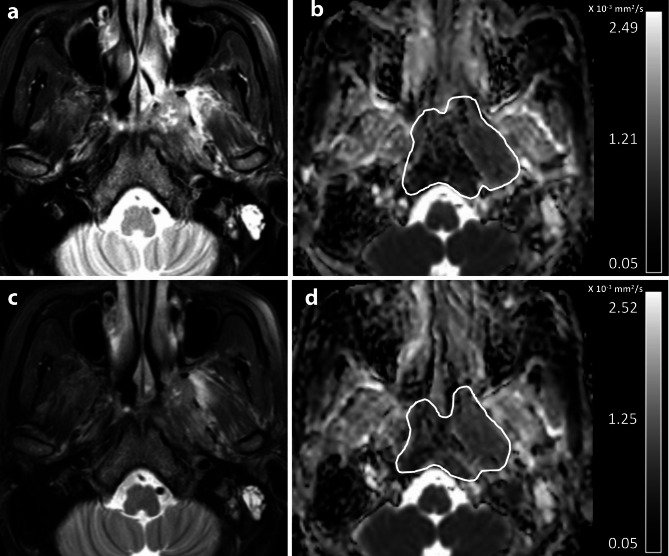
Pre-treatment (**a**-**b**) and post-IC (**c**-**d**) MRIs of a patient with stage T3 NPC that had recurrence 28 months after treatment. The axial images comprise T2-weighted fat-suppressed images (**a** and **c**) and ADC maps (**b** and **d**). The mean ADC_pre_ and ADC_post−IC_ extracted from the primary NPC (white contours) on the ADC maps were 0.89 × 10^− 3^ mm^2^/s, and 0.99 × 10^− 3^ mm^2^/s, respectively and the ΔADC% was 11.2%. The poor long-term outcome was predicted by a low percentage increase in mean ADC value (ΔADC% of < 34.2%) on the post-IC DWI compared with that on the pre-treatment DWI.

Table 2 shows results from the multivariable cox regression analysis for the correlation of ΔADC%, together with age, sex, T- and N-category, overall stage, cycles of chemotherapy with outcome. Results showed that the ΔADC% was an independent predictor of DFS, LRRFS, and DMFS (*p* < 0.01 to 0.03), greater ΔADC% predicting better outcome (Table 2). Furthermore, age was an independent predictor of DMFS, old age predicting poor DMFS (*p* = 0.01); cycles of IC was an independent predictor of DMFS, higher cycles of IC predicting better DFS and DMFS (both *p* < 0.01); and cycles of concurrent chemotherapy was an independent predictor of DMFS, higher cycles predicting better DMFS (*p* = 0.02) (Table 2). Other variables did not predict any of the outcome (*p* = 0.06 to 0.77) (Table 2). Two representative examples of the patient with adNPC who had no recurrence after treatment and who had local recurrence after treatment predicted by the ΔADC% are shown in Figs. 1 and 2, respectively.

**Table 2:** Multivariable Cox regression analysis for the correlations of significant measurement, patient demographics, cancer staging, and treatment details with outcome

	DFS		LRRFS		DMFS	
	HR (95%CI)	P-value	HR (95%CI)	P-value	HR (95%CI)	P-value
ΔADC%	0.945 (0.910–0.980)	**< 0.01**	0.887 (0.793–0.991)	**0.03**	0.923 (0.871–0.978)	**< 0.01**
Age	1.070 (0.994–1.152)	0.08	0.928 (0.804–1.070)	0.30	1.241 (1.061–1.451)	**0.01**
Sex (female as ref.)	1.926 (0.401–9.255)	0.41	2.935 (0.268–32.109)	0.40	5.051 (0.411–62.112)	0.21
T-category	2.458 (0.976–6.187)	0.06	2.887 (0.533–15.632)	0.22	0.923 (0.871–22.164)	0.08
N-category	1.828 (0.0.944–3.538)	0.08	0.757 (0.243–2.355)	0.63	2.447 (0.985–6.077)	0.06
Overall stage	0.168 (0.024–1.193)	0.08	1.644 (0.061–4.585)	0.77	0.560 (0.094–1.525)	0.09
Cycles of IC	0.115 (0.023–0.565)	**< 0.01**	7.110 (0.304–16.565)	0.22	0.110 (0.055–0.211)	**0.01**
Cycles of Concurrent chemotherapy	0.771 (0.527–1.129)	0.18	1.708 (0.675–4.324)	0.26	0.459 (0.245–0.858)	**0.02**

DFS = disease free survival, LRRFS = locoregional recurrence free survival, DMFS = distant metastases free survival, IC = induction chemotherapy, ADC = apparent diffusion coefficient

To examine whether ΔADC% being the independent variable for outcome was resulted from the overfit of the confounding variables in the multivariable analysis, we then performed the multivariable analysis by only fitting the ΔADC%, together with T- and N- categories, which are two confounding variables closely relate to long-term outcome in NPC. Results showed that ΔADC% was an independent predictor for DFS, LRRFS, and DMFS (*p* < 0.01 to 0.03) (Supplementary Table 1).

### Performances of RECIST groups and ΔADC% for the prediction of outcome

**Fig. 3:**
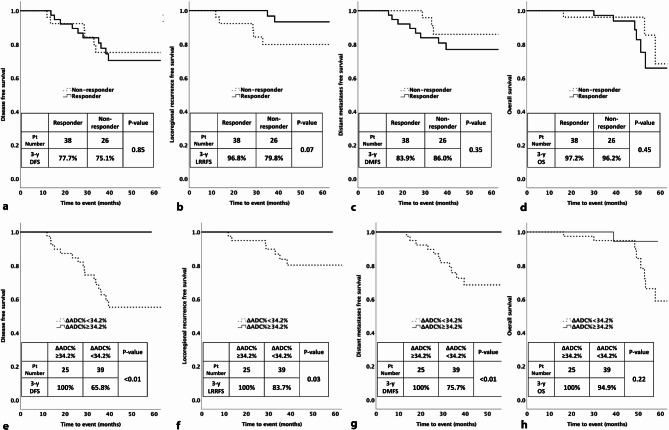
shows Kaplan-Meier curves of the RECIST groups (response and non-response) (**a**-**d**) and the ΔADC% groups (< 34.2% and ≥ 34.2%) (**e**-**h**) for the prediction of disease free survival (DFS) (**a** and **e**), locoregional recurrence free survival (LRRFS) (**b** and **f**), distant metastases free survival (DMFS) (**c** and **g**), and overall survival (OS) (**d** and **h**). Differences in all survivals between RECIST groups were not statistically significant (*p* = 0.07 to 0.85)(**a**-**d**) and that between the ΔADC% groups were statistically significant (*p* < 0.01 to 0.03), except that in OS (*p* = 0.22). Compared with those with RECIST responder, patients with a ΔADCV% of ≥ 34.2% showed a great in 3-year DFS (100% and 77.7%, respectively), in 3-year LRRFS (100% and 96.8%, respectively), in 3-year DMFS (100% vs. 83.9%, respectively) and in 3-year OS (100% and 97.2%, respectively

The optimal ΔADC% threshold using the maximised sensitivity and specificity for the prediction of disease recurrence was 34.2%. There were 39 patients categorised to ΔADC% < 34.2% and 25 categorised to ΔADC% ≥ 34.2% groups (ΔADC% groups). Table 3 shows the c-indexes of the ΔADC% groups and that of RECIST groups (response vs. non-response groups) for the prediction of DFS, LRRFS, DMFS, and OS. Compared with the RECIST groups, the ΔADC% groups showed a higher c-index for 3-year (c-index: 0.47 vs. 0.71, *p* < 0.01) and 5-year DFS (c-index: 0.51 vs. 0.72, *p* < 0.01) (Table 3), but there were no statistical differences in c-indexes between RECIST groups and the ΔADC% groups for 3-year and 5-year LRRFS, DMFS and OS (*p* = 0.13–0.89) (Table 3). For the RECIST groups, the Kaplan-Meier curves showed no statistical differences in DFS, LRRFS, DMFS and OS between responder and non-responder groups (*p* = 0.07–0.85) (Fig. 3). For the ΔADC% groups, compared with those with ΔADC% < 34.2%, patients with ΔADC% ≥ 34.2% had statistically higher 3-year DFS of 100% (*p* < 0.01), LRRFS of 100% (*p* = 0.03) and DMFS of 100% (*p* < 0.01), but there was no statistical difference in OS between ΔADC% groups (Fig. 3).

**Table 3:** C-index of the RECIST groups and ΔADC% groups for 3- and 5- year DFS, LRRFS, DMFS and OS

	DFS		LRRFS		DMFS		OS	
	C-index (95%CI)	P-value	C-index (95%CI)	P-value	C-index (95%CI)	P-value	C-index (95%CI)	P-value
3-year								
RECIST groups	0.47 (0.41–0.52)	Ref.	0.72 (0.60–0.85)	Ref.	0.54 (0.44–0.64)	Ref.	0.45 (0.33–0.58)	Ref.
ΔADC% groups	0.71 (0.68–0.75)	**< 0.01**	0.70 (0.66–0.73)	0.89	0.71 (0.67–0.74)	0.13	0.69 (0.63–0.75)	0.29
5-year								
RECIST groups	0.51 (0.45–0.58)	Ref.	0.69 (0.56–0.84)	Ref.	0.57 (0.48–0.66)	Ref.	0.53 (0.43–0.64)	Ref.
ΔADC% groups	0.72 (0.68–0.77)	**< 0.01**	0.70 (0.68–0.73)	0.78	0.71 (0.68–0.75)	0.22	0.60 (0.52–0.68)	0.68

RECIST groups = responder group vs. non-responder group

ΔADC% groups = ΔADC% < 34.2% group vs. ΔADC% ≥34.2% group

DFS = disease free survival, LRRFS = locoregional recurrence free survival, DMFS = distant metastases free survival, OS = overall survival, ADC = apparent diffusion coefficient

## Discussion

This study investigated the role of DWI obtained from the pre- and post-IC MRI scans for the prediction of long-term outcome in patients with adNPC. Our results showed that percentage change in ADC between pre- and post-IC scans (ΔADC%) was the only diffusion weighted parameter that correlated with DFS, LRRFS, and DMFS, high ΔADC% (greater % increase in ADC on the post-IC scan) predicting good outcome. Neither ADC_pre_ nor ADC_post−IC_ predicted survival using any of the endpoints in this study. After adjusting confounding factors (age, gender, T- and N-categories, overall stage, and treatment), ΔADC% remained independently predictive of DFS, LRRFS and DMFS. When compared with RECIST response groups (responder vs. non-responder), the ΔADC% groups which was grouped by using the optimal ΔADC% threshold of 34.2% improved the predictive values showing a c-index increased from 0.47 to 0.71 and from 0.51 to 0.72 for 3-year and 5-year DFS, respectively. The finding indicated a reduction in the restriction of water molecules in the primary tumor after IC is a stronger indicator of long-term outcome than shrinkage in size.

In previous HNSCC studies [10–16, 18, 21], ΔADC% has shown consistent predictive values for long-term outcome and so is the most promising one for clinical practice. In these HNSCC studies, most of studies only focused on the post-treatment locoregional recurrence because that the locoregional recurrence is the main cause of death in patients with HNSCC [11, 13–16]. For NPC, although many studies have showed ΔADC% is valuable for the prediction of short-term response evaluated within 6 months after treatment on the primary tumour bed [22–24, 36], only one focused on long-term outcome [25], which showed that ΔADC% is also valuable for the prediction of long-term outcome. Results from our study provided additional confirmation in that ΔADC% also predicted long-term outcome. In keeping with the trend of ADC change for the prediction of outcome reported previously [22–25, 36], our study showed that great ΔADC% (i.e. great increase in ADC on the post-IC scan) predicted better long-term outcome. One of the possible explanations is that anti-tumour treatment can result in tumour cell necrosis, which is reflected by the increase in ADC values, and great increase in ΔADC% may reflect tumour cells which are sensitive to treatment, thus being likely to response to treatment and be cured.

Several ΔADC% thresholds have been proposed in the previous head and neck cancer studies for the prediction of long-term outcome [13–16, 22–25]. These thresholds ranged widely (14 − 60%) possibly because of differences in prediction scenarios and time-intervals between pre- and inter-treatment DWI scans. Nevertheless, all of these proposed thresholds are similar to or above the intrinsic variability of DWI (about ΔADC% of 15%). In our study, we identified the optimal ΔADC% threshold of 34.2% by considering the predictive power for disease recurrence. We showed that patients with a ΔADC% of ≥ 34.2% had 100% survival rates for 3-year DFS, LRRFS, DMFS and OS, which indicated this group of patients did not have locoregional recurrence or distant metastases at least 3 years after the treatment. We believe that the ΔADC% of ≥ 34.2% could be clinically useful to confirm NPC patients, who do not need additional adjuvant chemotherapy or advanced treatment or close post-treatment follow-up. This group of patients would avoid complications and side effects from the unnecessarily additional treatment. Meanwhile, medical resources can be precisely allocated to other patients particularly in areas with limited resources. Nevertheless, it is worthy to note that the use of ΔADC% of ≥ 34.2% standard alone identified only about 50% of patients (25/48, 52%) who had no disease recurrence. There still has space to improve the performance to maximise the numbers of patients to benefit from the precise medical management. Therefore, other predictors that play complementary role to this DWI parameter are suggested to be included in the future clinical practice to identify patients at low risk of disease recurrence particularly from those who have ΔADC% of < 34.2%.

For patients with adNPC, the IC is routinely used to shrink the size of tumours to secure more critical tissues from the RT treatment and to diminish the micro disseminated tumour cells to decrease the risk of disease recurrence. The size of tumour shrinkage may be a predictive value for long-term outcome because tumour without shrinkage after IC may be resistant to treatment, thus being at risk of recurrence afterwards. Some previous head and neck cancer study investigated tumour responses to IC evaluated by using the RECIST guideline for the prediction of long-term outcome [37, 38]. In keeping with the results reported by Zeng et al. [38], our results showed that RECIST response groups did not predict outcome. However, conflicting results were reported [37]. The possibly explanation for the discrepancy is that the unidimensional measurement recommended by the RECIST guideline may not accurately reflect shrinkage of head and neck cancer during treatment.

In this study, we also investigated the predictive values of DWI on the pre-treatment scan for the prediction of long-term outcome. In keeping with our previous findings [39–41], this study showed that ADC_pre_ did not predict any of the long-term outcome, although some other studies showed conflicting results [42–44]. We further investigated predictive value of mean ADC on the post-IC for long-term outcome in NPC, which showed that absolute ADC_post−IC_ was not a predictor. Interestingly, we found that patients with old age were more likely to have distant metastases, possibly because that the biological degeneration of immune system in elderly increases the risk of residual tumour cells escaping from the self-immune surveillance. In terms of treatment, we showed that the increase in the cycles of chemotherapy predicted better outcome, probably due to the benefits from chemotherapy to decrease the risk of distant metastases. However, there have conflicting results been reported [45–48], possibly resulted from the retrospective design of the study. Therefore, further prospective studies dedicate to the investigation of the role of cycles of chemotherapy for outcomes are suggested.

This study has limitations. First, this study only included patients with adNPC and treated with IC + CCRT. The predictive value of ΔADC% for long-term outcome in patients who treated only by RT or CCRT remains unknown. Second, this study only focused on the assessment of the primary tumour, and metastatic nodes were not considered in the analysis. Third, although the median follow-up time of patients without relapse in this study was long (44.7 months), it is possible that a few patients may still relapse with a longer follow-up time. Fourth, the study was restricted to only one intra-treatment time point, and it is unknown if this is the optimal time for early DWI assessment. Fifth, this study did not include other recently proposed imaging-related methods, such as the volumetric analysis [27] to evaluate treatment response as unlike RECIST guideline, these methods are yet widely applied to clinical practice. Furthermore, we wish that our study could provide another option to accurately predict long-term outcome in adNPC in which volumetric analysis is time-consuming. Six, as this is one of the first NPC studies that investigated the predictive value of ΔADC% for long-term outcome, we only included patients who had the same DWI protocols performed in both pre- and post-IC MRIs to minimise the potential biases from the intrinsic variability of scanning protocols. Therefore, the generalisibility of findings from this study requires further external prospective validation studies to examine before ΔADC% is applied to clinical practice. Furthermore, plasma Epstein-Barr virus DNA levels were not included in the analysis because not all patients had this test routinely at different phases of treatment.

## Conclusion

Results suggested that percentage change in DWI on the pre-treatment and post-IC scan (ΔADC%) independently predicted long-term outcome and was superior to RECIST response groups for the prediction of treatment outcome in patients with adNPC. A great increase in ΔADC% (i.e. a great percentage of the increase of ADC values between pre- and post-IC MRIs) predicted good outcome and the ΔADC% achieved better performances for the prediction of DFS compared with RECIST response groups. A ΔADC% threshold of ≥ 34.2% may be of valuable for identifying patients without disease recurrence after treatment, who can potentially benefit from the reduced post-treatment surveillance and exemption of additional treatment (i.e. adjuvant chemotherapy).

## Electronic supplementary material

Below is the link to the electronic supplementary material.

Supplementary Material 1

## Data Availability

The datasets used and/or analysed during the current study are available from the corresponding author on reasonable request.

## Abbreviations

ADC: Apparent Diffusion Coefficient
CR: Complete Response
DFMS: Distant Metastases Free Survival
DFS: Disease Free Survival
DWI: Diffusion Weighted Imaging
LRRFS: Locoregional Recurrence Free Survival
NPC: Nasopharyngeal Carcinoma
OS: Overall Survival
PR: Partial Response
PD: Progressed Disease
SD: Stable Disease

## References

[1] Lee AWM, Ng WT, Chan LLK, et al. Evolution of treatment for nasopharyngeal cancer - Success and setback in the intensity-modulated radiotherapy era. Radiother Oncol. 2014;110:377–84. 10.1016/j.radonc.2014.02.003. 24630534 10.1016/j.radonc.2014.02.003

[2] Lam WKJ, King AD, Miller JA, et al. Recommendations for Epstein-Barr virus–based screening for nasopharyngeal cancer in high- and intermediate-risk regions. J Natl Cancer Inst. 2023;115:355–64. 10.1093/jnci/djad012. 36723440 10.1093/jnci/djad012 PMC10086631

[3] Wong KCW, Hui EP, Lo KW, et al. Nasopharyngeal carcinoma: an evolving paradigm. Nat Rev Clin Oncol. 2021;18:679–95. 10.1038/s41571-021-00524-x. 34194007 10.1038/s41571-021-00524-x

[4] Lee AWM, Ma BBY, Ng WT, Chan ATC. Management of nasopharyngeal carcinoma: current practice and future perspective. J Clin Oncol. 2015;33:3356–64. 10.1200/JCO.2015.60.9347. 26351355 10.1200/JCO.2015.60.9347

[5] Thoeny HC, De Keyzer F, King AD. Diffusion-weighted MR imaging in the head and neck. Radiology. 2012;263:19–32. 10.1148/radiol.11101821. 22438440 10.1148/radiol.11101821

[6] Connor S, Christoforou A, Touska P, et al. An international survey of diffusion and perfusion magnetic resonance imaging implementation in the head and neck. Eur Radiol. 2025. 10.1007/s00330-025-11370-1. 39904786 10.1007/s00330-025-11370-1 PMC12226617

[7] Zhang Y, Luo D, Guo W, et al. Utility of mono-exponential, bi-exponential, and stretched exponential signal models of intravoxel incoherent motion (IVIM) to predict prognosis and survival risk in laryngeal and hypopharyngeal squamous cell carcinoma (LHSCC) patients after chemoradiotherapy. Jpn J Radiol. 2023;41:712–22. 10.1007/s11604-023-01399-x. 36847996 10.1007/s11604-023-01399-x PMC10313558

[8] Connor S, Sit C, Anjari M, et al. The ability of post-chemoradiotherapy DWI ADCmean and 18F-FDG SUVmax to predict treatment outcomes in head and neck cancer: impact of human papilloma virus oropharyngeal cancer status. J Cancer Res Clin Oncol. 2021;147:2323–36. 10.1007/s00432-021-03662-y. 34159420 10.1007/s00432-021-03662-y PMC8236463

[9] Guo W, Luo D, Lin M, et al. Pretreatment intra-voxel incoherent motion diffusion-weighted imaging (IVIM-DWI) in predicting induction chemotherapy response in locally advanced hypopharyngeal carcinoma. Med (United States). 2016;95. 10.1097/MD.0000000000003039. 10.1097/MD.0000000000003039 PMC4998905 26962824

[10] Wong KH, Panek R, Welsh L, et al. The predictive value of early assessment after 1 cycle of induction chemotherapy with 18F-FDG PET/CT and diffusion-weighted MRI for response to radical chemoradiotherapy in head and neck squamous cell carcinoma. J Nucl Med. 2016;57:1843–50. 10.2967/jnumed.116.174433. 27417648 10.2967/jnumed.116.174433

[11] King AD, Mo FKF, Yu K-H, et al. Squamous cell carcinoma of the head and neck: diffusion-weighted MR imaging for prediction and monitoring of treatment response. Eur Radiol. 2010;20:2213–20. 10.1007/s00330-010-1769-8. 20309553 10.1007/s00330-010-1769-8

[12] King AD, Chow K-K, Yu K-H, et al. Head and neck squamous cell carcinoma: diagnostic performance of Diffusion-weighted MR imaging for the prediction of treatment. Radiology. 2013;266:531–8. 23151830 10.1148/radiol.12120167

[13] Vandecaveye V, Dirix P, De Keyzer F, et al. Predictive value of diffusion-weighted magnetic resonance imaging during chemoradiotherapy for head and neck squamous cell carcinoma. Eur Radiol. 2010;20:1703–14. 10.1007/s00330-010-1734-6. 20179939 10.1007/s00330-010-1734-6

[14] Matoba M, Tuji H, Shimode Y, et al. Fractional change in apparent diffusion coefficient as an imaging biomarker for predicting treatment response in head and neck cancer treated with chemoradiotherapy. Am J Neuroradiol. 2014;35:379–85. 10.3174/ajnr.A3706. 24029391 10.3174/ajnr.A3706 PMC7965773

[15] Trada Y, Keall P, Jameson M, et al. Changes in serial multiparametric MRI and FDG-PET/CT functional imaging during radiation therapy can predict treatment response in patients with head and neck cancer. Eur Radiol. 2023. 10.1007/s00330-023-09843-2. 37405500 10.1007/s00330-023-09843-2 PMC10667402

[16] Vandecaveye V, Dirix P, De Keyzer F, et al. Diffusion-weighted magnetic resonance imaging early after chemoradiotherapy to monitor treatment response in head-and-neck squamous cell carcinoma. Int J Radiat Oncol Biol Phys. 2012;82:1098–107. 10.1016/j.ijrobp.2011.02.044. 21514067 10.1016/j.ijrobp.2011.02.044

[17] Tsuchiya H, Matoba M, Nishino Y, et al. Clinical utility of combined assessments of 4D volumetric perfusion CT, diffusion-weighted MRI and 18F-FDG PET-CT for the prediction of outcomes of head and neck squamous cell carcinoma treated with chemoradiotherapy. Radiat Oncol. 2023;18. 10.1186/s13014-023-02202-x. 10.1186/s13014-023-02202-x PMC9901150 36747228

[18] Brenet E, Barbe C, Hoeffel C, et al. Predictive value of early post-treatment diffusion-weighted mri for recurrence or tumor progression of head and neck squamous cell carcinoma treated with chemo-radiotherapy. Cancers (Basel). 2020;12. 10.3390/cancers12051234. 10.3390/cancers12051234 PMC7281260 32422975

[19] Noij DP, Pouwels PJW, Ljumanovic R, et al. Predictive value of diffusion-weighted imaging without and with including contrast-enhanced magnetic resonance imaging in image analysis of head and neck squamous cell carcinoma. Eur J Radiol. 2015;84:108–16. 10.1016/j.ejrad.2014.10.015. 25467228 10.1016/j.ejrad.2014.10.015

[20] Lambrecht M, Van Calster B, Vandecaveye V, et al. Integrating pretreatment diffusion weighted MRI into a multivariable prognostic model for head and neck squamous cell carcinoma. Radiother Oncol. 2014;110:429–34. 10.1016/j.radonc.2014.01.004. 24630535 10.1016/j.radonc.2014.01.004

[21] Berrak S, Chawla S, Kim S, et al. Diffusion weighted imaging in predicting progression free survival in patients with squamous cell carcinomas of the head and neck treated with induction chemotherapy. Acad Radiol. 2011;18:1225–32. 10.1016/j.acra.2011.06.009. 21835649 10.1016/j.acra.2011.06.009 PMC3168957

[22] Hong J, Yao Y, Zhang Y, et al. Value of magnetic resonance diffusion-weighted imaging for the prediction of radiosensitivity in nasopharyngeal carcinoma. Otolaryngol - Head Neck Surg (United States). 2013;149:707–13. 10.1177/0194599813496537. 10.1177/0194599813496537 23884282

[23] Chen Y, Ren W, Zheng D, et al. Diffusion kurtosis imaging predicts neoadjuvant chemotherapy responses within 4 days in advanced nasopharyngeal carcinoma patients. J Magn Reson Imaging. 2015;42:1354–61. 10.1002/jmri.24910. 25873208 10.1002/jmri.24910

[24] Tangyoosuk T, Lertbutsayanukul C, Jittapiromsak N. Utility of diffusion-weighted magnetic resonance imaging in predicting the treatment response of nasopharyngeal carcinoma. Neuroradiol J. 2022;35:477–85. 10.1177/19714009211055191. 34730049 10.1177/19714009211055191 PMC9437492

[25] Liu LT, Guo SS, Li H, et al. Percent change in apparent diffusion coefficient and plasma EBV DNA after induction chemotherapy identifies distinct prognostic response phenotypes in advanced nasopharyngeal carcinoma. BMC Cancer. 2021;21. 10.1186/s12885-021-09063-1. 10.1186/s12885-021-09063-1 PMC8662833 34886807

[26] Eisenhauer EA, Therasse P, Bogaerts J, et al. New response evaluation criteria in solid tumours: revised RECIST guideline (version 1.1). Eur J Cancer. 2009;45:228–47. 10.1016/j.ejca.2008.10.026. 19097774 10.1016/j.ejca.2008.10.026

[27] Kwong TSA, Leung HS, Mo FKF, et al. Volumetric measurement to evaluate treatment response to induction chemotherapy on MRI outperformed RECIST guideline in outcome prediction in advanced nasopharyngeal carcinoma. ESMO Open. 2024;9:103933. 10.1016/j.esmoop.2024.103933. 39368415 10.1016/j.esmoop.2024.103933 PMC11490768

[28] Ai QY, King AD, Chan JSM, et al. Distinguishing early-stage nasopharyngeal carcinoma from benign hyperplasia using intravoxel incoherent motion diffusion-weighted MRI. Eur Radiol. 2019;29:5627–34. 10.1007/s00330-019-06133-8. 30903340 10.1007/s00330-019-06133-8

[29] So TY, Ai QYH, Lam WKJ, et al. Intravoxel incoherent motion diffusion-weighted imaging for discrimination of benign and malignant retropharyngeal nodes. Neuroradiology. 2020;62:1667–76. 10.1007/s00234-020-02494-w. 32676831 10.1007/s00234-020-02494-w

[30] Li Q, Jiang T, Wang T, et al. Improved Readout-Segmented Echo-Planner Diffusion-Weighted magnetic resonance imaging of nasopharyngeal carcinoma using simultaneous multislice acquisitions at 3 T. J Comput Assist Tomogr. 2022;46:815–22. 10.1097/RCT.0000000000001327. 35483083 10.1097/RCT.0000000000001327 PMC9477861

[31] Zhang GY, Wang YJ, Liu JP et al. (2015) Pretreatment diffusion-weighted MRI can predict the response to neoadjuvant chemotherapy in patients with nasopharyngeal carcinoma. Biomed Res Int 2015. 10.1155/2015/307943 10.1155/2015/307943 PMC4564581 26413513

[32] Harrell FE, Lee KL, Mark DB. Tutorial in biostatistics multivariable prognostic models: issues in developing models, evaluating assumptions and adequacy, and measuring and reducing errors. Stasistics Med. 1996;15:361–87. 10.1002/(SICI)1097-0258(19960229)15:4<361::AID-SIM168>3.0.CO;2-4 8668867

[33] Uno H, Cai T, Pencina MJ, et al. On the C-statistics for evaluating overall adequacy of risk prediction procedures with censored survival data. Stat Med. 2011;30:1105–17. 10.1002/sim.4154. 21484848 10.1002/sim.4154 PMC3079915

[34] Correa AF, Jegede O, Haas NB, et al. Predicting renal Cancer recurrence: defining limitations of existing prognostic models with prospective Trial-Based validation. J Clin Oncol. 2019;37:2062–71. 10.1200/JCO.19. 31216227 10.1200/JCO.19.00107 PMC7085167

[35] Moons KGM, Altman DG, Reitsma JB, et al. Transparent reporting of a multivariable prediction model for individual prognosis or diagnosis (TRIPOD): explanation and elaboration. Ann Intern Med. 2015;162:W1–73. 10.7326/M14-0698. 25560730 10.7326/M14-0698

[36] Ai QYH, King AD, Tsang YM, et al. Predictive markers for head and neck cancer treatment response: T1rho imaging in nasopharyngeal carcinoma. Eur Radiol. 2024. 10.1007/s00330-024-10948-5. 39191996 10.1007/s00330-024-10948-5 PMC11836102

[37] Peng H, Chen L, Zhang Y, et al. The tumour response to induction chemotherapy has prognostic value for Long-Term survival outcomes after Intensity-Modulated radiation therapy in nasopharyngeal carcinoma. Sci Rep. 2016;6:24835. 10.1038/srep24835. 27099096 10.1038/srep24835 PMC4838936

[38] Zeng Y-Y, Xiang Z-Z, He T, et al. The comparison of prognostic value of tumour volumetric regression ratio and RECIST 1.1 criteria after induction chemotherapy in locoregionally advanced nasopharyngeal carcinoma. Oral Oncol. 2020;111:104924. 10.1016/j.oraloncology.2020.104924. 32736209 10.1016/j.oraloncology.2020.104924

[39] Ai Q-Y, King AD, Law BKH, et al. Diffusion-weighted imaging of nasopharyngeal carcinoma to predict distant metastases. Eur Arch Otorhinolaryngol. 2017;274:1045–52. 10.1007/s00405-016-4333-6. 27722898 10.1007/s00405-016-4333-6

[40] Law BKH, King AD, Bhatia KS, et al. Diffusion-Weighted imaging of nasopharyngeal carcinoma: can pretreatment DWI predict local failure based on Long-Term outcome?? Am J Neuroradiol. 2016;37:1706–12. 10.3174/ajnr.A4792. 27151750 10.3174/ajnr.A4792 PMC7984690

[41] Qamar S, King AD, Ai QYH, et al. Pre-treatment intravoxel incoherent motion diffusion-weighted imaging predicts treatment outcome in nasopharyngeal carcinoma. Eur J Radiol. 2020;129:109127. 10.1016/j.ejrad.2020.109127. 32563165 10.1016/j.ejrad.2020.109127

[42] Yan DF, Zhang WB, Ke SB, et al. The prognostic value of pretreatment tumor apparent diffusion coefficient values in nasopharyngeal carcinoma. BMC Cancer. 2017;17. 10.1186/s12885-017-3658-x. 10.1186/s12885-017-3658-x PMC5637091 29020937

[43] Huang TX, Lu N, Lian SS, et al. The primary lesion apparent diffusion coefficient is a prognostic factor for locoregionally advanced nasopharyngeal carcinoma: A retrospective study. BMC Cancer. 2019;19. 10.1186/s12885-019-5684-3. 10.1186/s12885-019-5684-3 PMC6525458 31101029

[44] Zhang Y, Liu X, Zhang Y, et al. Prognostic value of the primary lesion apparent diffusion coefficient (ADC) in nasopharyngeal carcinoma: a retrospective study of 541 cases. Sci Rep. 2015;5:12242. 26184509 10.1038/srep12242 PMC4505330

[45] Ahmed AO, Wang J, Wu Q, Zhong Y. Determination of optimum number of cycles of induction chemotherapy for locoregionally advanced nasopharyngeal carcinoma: a single-center retrospective study. Eur Arch Otorhinolaryngol. 2023;280:1999–2006. 10.1007/s00405-022-07794-w. 36629931 10.1007/s00405-022-07794-w

[46] Zhan Z, Huang Y, Zhou J, et al. Integrated strategies for chemotherapy cycles in nasopharyngeal carcinoma patients: Real-world data from two epidemic centers guiding decision-making. Chin J Cancer Res. 2023;35:126–39. 10.21147/j.issn.1000-9604.2023.02.04. 37180835 10.21147/j.issn.1000-9604.2023.02.04 PMC10167606

[47] Zhao X, Tian L, Chen Y, et al. Long-term outcomes of induction chemotherapy followed by concurrent chemoradiotherapy and adjuvant chemotherapy for locoregionally advanced nasopharyngeal carcinoma: a retrospective study. Front Oncol. 2024;14. 10.3389/fonc.2024.1475176. 10.3389/fonc.2024.1475176 PMC11632233 39664180

[48] He Y, Zhao Z, Wang Y, et al. Optimizing number of cycles of induction chemotherapy for patients with nasopharyngeal carcinoma: retrospective survival analysis. Head Neck. 2020;42:2067–76. 10.1002/hed.26141. 32202686 10.1002/hed.26141

