# Supplementary material for: Change in diffusion weighted imaging after induction chemotherapy outperforms RECIST guideline for long-term outcome prediction in advanced nasopharyngeal carcinoma
Source: Cancer Imaging. 2025 Mar 12;25:32. doi: 10.1186/s40644-025-00854-4 (PMC11905565; doi:10.1186/s40644-025-00854-4)
Supplement: Supplementary file 1 — Supplementary Material 1 [file 40644_2025_854_MOESM1_ESM.docx]

Supplementary Table 1. Multivariable Cox regression analysis for the correlations of significant measurement and T- and N- categories with outcomes

|  | **DFS** | | **LRRFS** | | **DMFS** | |
| --- | --- | --- | --- | --- | --- | --- |
|  | HR  (95%CI) | P-value | HR  (95%CI) | P-value | HR  (95%CI) | P-value |
| ΔADC% | 0.957  (0.929 – 0.985) | **<0.01** | 0.930  (0.874 – 0.990) | **0.02** | 0.964  (0.933 – 0.997) | **0.03** |
| T-category | 1.636  (0.896 – 2.986) | 0.11 | 1.726  (0.566 – 5.266) | 0.34 | 1.611  (0.772 – 3.363) | 0.20 |
| N-category | 1.300  (0.737 – 2.294) | 0.37 | 0.780  (0.335 – 1.815) | 0.56 | 1.313  (0.652 – 2.646) | 0.45 |

DFS =disease free survival, LRRFS = locoregional recurrence free survival, DMFS = distant metastases free survival, ADC =apparent diffusion coefficient
